# Do Peri-Urban Areas Act as Refuges for the European Rabbit (*Oryctolagus cuniculus* L.)?

**DOI:** 10.3390/ani15182719

**Published:** 2025-09-17

**Authors:** Ana Cabrera, Cristina Mata, Juan E. Malo

**Affiliations:** 1 Terrestrial Ecology Group (TEG-UAM), Departamento de Ecología, Universidad Autónoma de Madrid, 28049 Madrid, Spaincristina.mata@uam.es (C.M.); 2 Centro de Investigación en Biodiversidad y Cambio Global (CIBC-UAM), Universidad Autónoma de Madrid, 28049 Madrid, Spain

**Keywords:** fragmentation, habitat selection, land-use planning, landscape of fear, Green Infrastructure, predation, road, urban ecology, urban encroachment

## Abstract

The European rabbit (*Oryctolagus cuniculus*) is a key species in many Mediterranean ecosystems, especially as prey for numerous Iberian predators. Due to its population decline and spatially heterogeneous recovery following different diseases, there is a need to understand the factors driving local abundance, and a potential refuge effect of anthropized sites has been speculated. In this study, 18 peri-urban areas in Central Spain were selected and a rabbit abundance index was measured in those areas and in parallel controls. Urban occupation and vegetation cover around sampling points were measured, as well as distance to roads, forest patches, and rivers, as factors which can drive rabbit abundance. Linear models were built to unravel general trends in data, from the perspectives of vegetation vs. human occupation and larger landscape-scale effects on rabbit abundance. Negative effects of urban development, distance to roads and proportion of pastures on rabbit abundance were identified, in addition to a positive effect of distance to large forest patches. Regarding urbanization, differences were found among urban types, with a significant negative effect of single-family residential occupation in comparison with multi-family and commercial/industrial occupation. In short, urbanization affects rabbit density, but it does not produce a generalized refuge area surrounding human settlements.

## Simple Summary

The European rabbit is a key species in the Iberian Peninsula, as well as invasive in many areas around the world. However, European rabbit populations declined due to different diseases in its native area, and several Iberian predators strongly suffered from that. Now, rabbit populations are increasing, but in a patchy way, and the reasons for that are not clear. Surprisingly, rabbits have become rather abundant close to several roads, railways, and villages, and it has been posed that peri-urban sites may act as refuges for them. Here we analyze the case in 18 municipalities in Madrid Region. We sampled rabbit abundance in empty lots surrounding different types or urban areas as well as distant control sites, and we analyze the relevance of factors associated with human occupation, plant cover, and landscape for the abundance of the species. We find that human factors are relevant for the species, but urbanism has a mixed effect, resulting in a reduced abundance of rabbits around single-family residential areas. Thus, human activity may facilitate the local development of dense rabbit populations due to interference with predators, but free-ranging pets and predators living in neighborhoods with large gardens may reverse this situation.

## 1. Introduction

Earth’s ecosystems are extremely modified by human activities, to the point of approaching the limits of safety for resilient and human-adequate function in the mid-term future [1,2]. Linked to these changes, biological communities are greatly disturbed and increasingly dependent on their interaction with humans [3,4]. At the same time, human interaction with wildlife is increasing, as humanity steadily concentrates in urban areas close to forests and other natural areas [5,6], and the structure of cities changes from compact forms to combinations of dense urban centers and residential areas with sparse houses among gardens, large parks, and unoccupied spaces [7]. In parallel, other infrastructure such as roads, dams, and mines multiply across the territory to meet human needs, extending the human footprint to all types of ecosystems [8]. Under these conditions, the number of endangered species increases, and the survival of many of them relies on their ability to coexist more or less closely with these activities [9,10]. Secondarily, the (global or local) species extinction and the favoring of certain species tolerant to humans leads to an impoverishment and simplification of biotic communities [11]. Thus, to advance towards sustainability we need to understand better the interactions between animal species and human activities, since wildlife survival will increasingly depend on our ability to minimize human impact on it.

Urban areas, and vertebrates associated with them, are a particular case and of utmost interest in this situation, both because of the human tendency to concentrate in cities and the stated biodiversity crisis [12,13]. Urbanization imposes changes to the living conditions of species, acting as a filter, so that vertebrate communities in urbanized areas become simplified and the abundance of generalist species tolerant to human increases [14,15]. In turn, such changes in biophysical conditions and in the species inhabiting cities lead to modifications in ecological processes [16,17], among which predator–prey interactions have attracted scientific attention in recent decades. In general, it has been found that predation pressure is reduced in cities because, although predator density may be high, the absence of apex predators and the parallel increase in opportunistic predators reduces effective predation pressure and redirects it toward a human-subsidized commensalism supported on garbage, pet food, fruits from garden plants and many other edible items [18,19]. Consequently, some prey species with a certain tolerance to humans can establish stable and relatively abundant populations in urban and peri-urban areas by three complementary processes. Namely, these are (i) a reduction in predator numbers (due to avoidance or persecution), (ii) a reduced accessibility to the territory due to fragmentation, or (iii) their shift in diet. In such situations, urban and peri-urban areas can provide local refuges for prey species [20].

In this context, the European rabbit (*Oryctolagus cuniculus*) is of great interest, due to its key role in ecosystems and because its populations are sometimes associated with human activities. The rabbit has been defined as a key species in the Iberian Peninsula (reviewed in [21]) due to its ability to consume and disperse a wide variety of plant species, its burrowing and soil disturbance activity, and most notably due to its role as the main prey of several predators. Thus, in the Iberian Peninsula, where it is native, it is consumed by more than forty predator species, many of which are endangered and are the focus of conservation programs [21,22]. The rabbit is currently classified as endangered by the IUCN in its natural range due to the drastic population decline, mainly as a result of two viral diseases: myxomatosis and rabbit hemorrhagic disease [23]. Land-use changes also pose a threat to the species [24,25], so recovery plans include promoting mosaics of forest, scrub, and grassland where the species reaches high densities [26,27]. These mosaics provide the ideal combination of food and shelter for rabbits, as the population dynamics of the species are highly dependent on the intensity of predation (or hunting) suffered by their populations [28].

At the same time, rabbits have high reproductive potential and a great capacity to invade, often developing dense populations that conflict with human activities and infrastructure. In fact, the species has been introduced to every continent, and it is considered one of the 100 most harmful invasive species in the world due to the damage it causes to crops and human infrastructure [22,29]. Among the human infrastructures where the species is most abundant are roads, where they proliferate along verges taking advantage of the availability of vegetation, diggable soil, and lower predation pressure [30,31]. In fact, on the Iberian Peninsula, rabbits cause damage to crops mainly where roads or railways cross agrarian plains [32], and the company managing railways spends large sums of money to prevent or correct damage to the infrastructure [33].

In urban areas, rabbits establish permanent populations in parks, gardens, and other places with diggable soil, sometimes with densities higher than those recorded in the surroundings [12,34]. Urban development facilitates the establishment of rabbit populations in different ways. On the one hand, urban areas and their surroundings are free from hunting, and the abundance of predators can be reduced in them [35] (see however [36]). In addition, the soil is not plowed, and there are gardens and empty lots where rabbits can settle permanent burrows and enjoy relative tranquility [34,37]. On the other hand, the presence of free-roaming pets poses a real and/or perceived threat to them, with potential negative effects on their establishment and demographic parameters [38,39]. Finally, urban and peri-urban areas have a high density of streets and roads of various types with surfaces covered by vegetation, like verges and roundabouts, potentially hosting rabbits (see above), but intense traffic can cause additional mortality to them [40]. In short, urban and peri-urban areas can create a refuge effect for rabbits depending on the net balance between the opportunities they offer (food, stable soils for burrows, etc.) and the risks they pose (roadkill, predation, etc.), facilitating the development of dense populations where the former outweigh the latter [14,41]. This balance can vary between types of human occupation, so that along the rural–urban gradient, the balance may shift (e.g., free-roaming cats and walkers with dogs are more common in the outskirts of single-family residential areas [42,43].

In this context, the objective of this study is to evaluate whether undeveloped land lots surrounding towns act as refuges, resulting in a greater abundance of rabbits there than further away from urbanization. Our starting hypothesis is that this is a widespread process, and therefore an increased abundance of rabbits will be found in a buffer around most villages within the distribution area of the species. In addition, other factors that may modulate the local abundance of rabbits are analyzed, such as (i) the type of plant cover in the immediate environment (within its home range), which determines the availability of food and shelter, and (ii) landscape features on a larger scale (distance to rivers, forests, and roads), which may determine the presence of predators. Finally, (iii) we explore the possibility that the type of urbanism (multi-family neighborhood, single-family residential area, or commercial/industrial) influences the local abundance of the species in peri-urban areas.

## 2. Materials and Methods

### 2.1. Study Area and Site Selection

**Figure 1:**
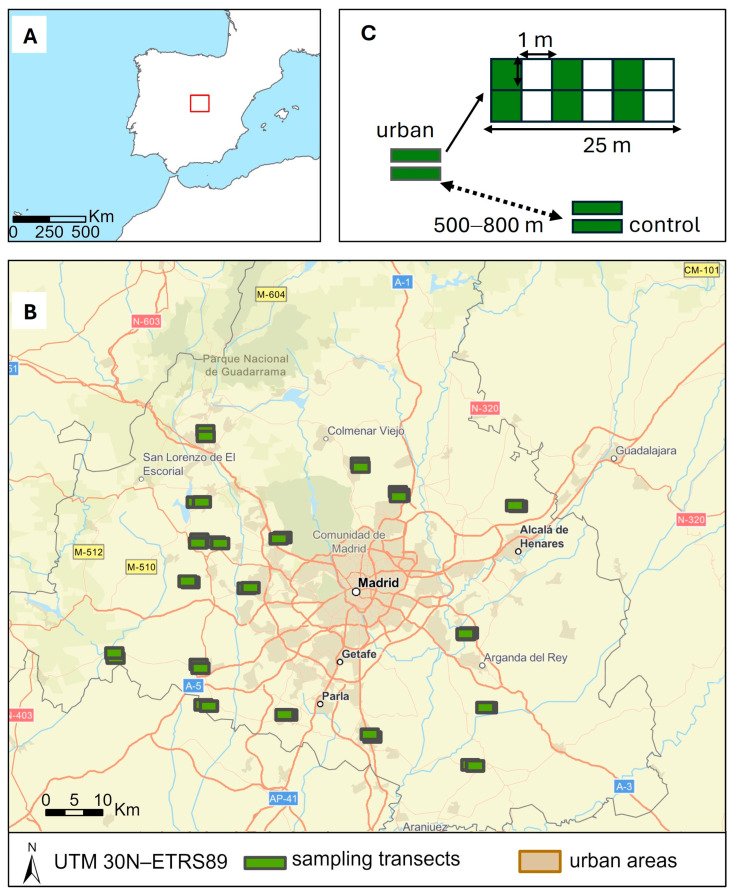
Location of the study area in the context of the Iberian Peninsula (**A**) and of sampling localities within it (**B**). As shown in (**C**), at each locality a peri-urban and a control site were selected, and the abundance of rabbit pellets sampled in two 25 × 2 m transects in each of them. Rabbit pellets were estimated visually in each of their 1 × 1 m grid cells to produce an aggregated index of pellet abundance in 50 m^2^ (see text).

The study was conducted in the south–central area of the Madrid Region (Central Spain), covering a region of approximately 5400 km^2^ located between 450 and 900 m above sea level and with conditions suitable for the development of the species (Figure 1A,B). Thus, the entire area has diggable soils, a dry Mediterranean climate, and mixtures of grassland, scrubland, and sclerophyllous forest habitats. In fact, Madrid is one of the regions defined as having the highest density of rabbits on the Iberian Peninsula [44] even though it harbors the capital of the country and over 7 million inhabitants.

Within this area, a blind search was conducted for locations where peri-urban areas could be sampled, with the aim of ensuring that they were representative and met the following criteria: (i) presence of patches adjacent to built-up areas where traditional agricultural activity (mainly plowing) had ceased, (ii) distribution spread throughout the area, (iii) existence of potential control sampling points with a similar landscape structure located at distances of 500–800 m away from the urbanized edge, and (iv) representative of different types of urbanism (from rural to industrial and urban) and types of landscape (from open to more forested ones). To conduct this search, we worked with official online vegetation and urban planning map repositories [45,46], supporting our searches with 2021 aerial photography with 25 cm pixel resolution [45]. Once the potential sampling sites had been selected, they were visited to confirm their accessibility for sampling, and if they were not viable, they were replaced by new points using the procedure explained above. This protocol was followed to minimize possible location biases associated with prior knowledge of local rabbit abundances. A total of 18 sampling localities were finally defined using this procedure (Figure 1B).

### 2.2. Rabbit Sampling and Explanatory Variables

Between March and May 2022, field sampling was carried out using an abundance index based on pellet counts as an approximation of rabbit abundance [47,48]. At each locality, two sampling sites were selected (a peri-urban site and a control site 500–800 m away), and at each of these sites, two parallel 2 × 25 m transects were established, separated by 50 m. In each 50 m^2^ transect, rabbit droppings were estimated for every 1 × 1 m grid within it (Figure 1C). In each grid, a visual estimate of the number of droppings present was made, using the methodology proposed in [49], and from these, the relative abundance index (RAI) of droppings for the transect was calculated as the sum of the fifty median values Ci (Table 1). For all analyses, the average value of each pair of transects is used as response variable.

Each sampling site was characterized in terms of variables that could potentially explain rabbit abundance at two different spatial scales: (i) a 300 m buffer around the central point between the sampling transects, reflecting the home range conditions of the rabbits present at the sampling point [26], and (ii) a larger area that could explain effects at broader landscape scales. For the buffer area, the occupation by human activities was measured on aerial images in three classes, and vegetation cover in six classes according to the SIGPAC classification (Table 2, [50]). The three types of urbanism correspond to a gradient of intensity of human transformation of the environment with a potential effect on biological communities [51]. The six categories of vegetation cover reflect different conditions of food and shelter availability that have been described as key to the abundance of wild rabbits [28,52], and they were calculated here as a percentage cover of the area not occupied by any type of urban development. The presence of transport infrastructure (roads and railways), permanent rivers, and forest patches larger than 5 ha were taken into account as landscape features at a larger scale with potential impact on rabbits. The presence of transport infrastructure drives the emergence of high-density rabbit populations [32], and patches of forest and permanent rivers represent places where mesocarnivores frequently inhabit humanized landscapes [53,54]. For each of the three cases, the minimum distance from the center point of each sampling site to the nearest element of interest was measured using GIS. All geographic information was managed in QGIS [55].

### 2.3. Data Analysis

First, an exploratory analysis of variables was performed, which led to the logarithmic transformation of the response variable as well as the distances to transport infrastructures, forest patches, and rivers, to improve their variance-to-average ratios. Furthermore, no correlations greater than 0.7 were found between explanatory variables, with the sole exception of the (expected) relationship between the peri-urban/control factor and the percentage of urbanized area within the 300 m buffer. For this reason, the aforementioned factor was not included in models containing the urbanized area (see below).

To analyze the effect of urbanization and other factors determining rabbit abundance, a sequential approach was taken using hypothesis-driven general linear mixed models. With this aim, three initial models were defined that could potentially explain the abundance of rabbits, corresponding to (i) human activity factors, represented by the percentage of urbanized land and the distance to transport infrastructure; (ii) landscape structure within the rabbit home range, represented by the percentage of cover of different types of vegetation in the buffer area not occupied by urban uses; and (iii) landscape factors at a wider scale which may drive the presence of predators in the area, namely distance to forest patches and distance to permanent rivers. In models ii and iii, the peri-urban/control factor was also included, and in all three models, the random factor ‘sampling location’ was introduced. Each model was simplified, and the best model was selected according to Akaike’s Information Criterion (AIC). By this means we generated the shortlist of variables potentially explaining rabbit abundance in the study area.

Finally, a general model was built based on the variables selected in the previous step, with the variables to be retained evaluated again by their informative capacity according to AIC. Moreover, in this phase (i) the relevance of the random factor locality was verified, in order to further simplify the model to a general linear model in the case of no relevance of the locality; and (ii) the relevance of different types of urbanism was explored by introducing as explanatory variables the percentages of cover by single-family residential, commercial/industrial, and multi-family residential areas, instead of that of total urbanized cover. All analyses were performed with AICModavg, car, lme4, lmerTest, MASS, and system libraries in R software environment (version 4.5.1) [56].

## 3. Results

The results indicate the existence of a large spatial variability in the abundance of rabbits, and a tendency for them to be more abundant in the control points than in the peri-urban areas. Thus, abundance indices for control points reached higher values (RAI mean ± SD: 2401.0 ± 2191.7 pellets/50 m^2^, range: 0–7238) than those obtained at points closer to urban areas (RAI mean ± SD: 1402.0 ± 1780.9 pellets/50 m^2^, range: 0–6978).

The selection of explanatory variables for the relative abundance of rabbits showed that it is associated with the three types of hypothesized effects, with variables corresponding to anthropogenic factors, habitat structure at the home range scale, and landscape structure at larger scales shaping it. Thus, the best model related to the effects of human activity (AIC = 167.2) retains both urban cover and distance to transport infrastructure as explanatory variables. Specifically, the abundance of rabbits increases with proximity to infrastructure and decreases with the percentage of urbanized cover. In relation to vegetation cover at the home range scale, the best model (AIC = 174.4) indicates that rabbit abundance decreases with increasing grassland cover, and it is indifferent to whether the sampling point is peri-urban or control. Finally, the best model of variables measured at the landscape scale (AIC = 171.9) retains as explanatory variables the positive effect on rabbit abundance of distance from forest patches and the existence of lower rabbit abundance in peri-urban areas. The construction of a joint model with these variables (AIC = 164.3) shows that all the explanatory variables selected above are informative, with the model without random factor being better (ΔAIC = −6.04) and there being no plausible alternative model (ΔAIC > 2 in all cases).

**Figure 2:**
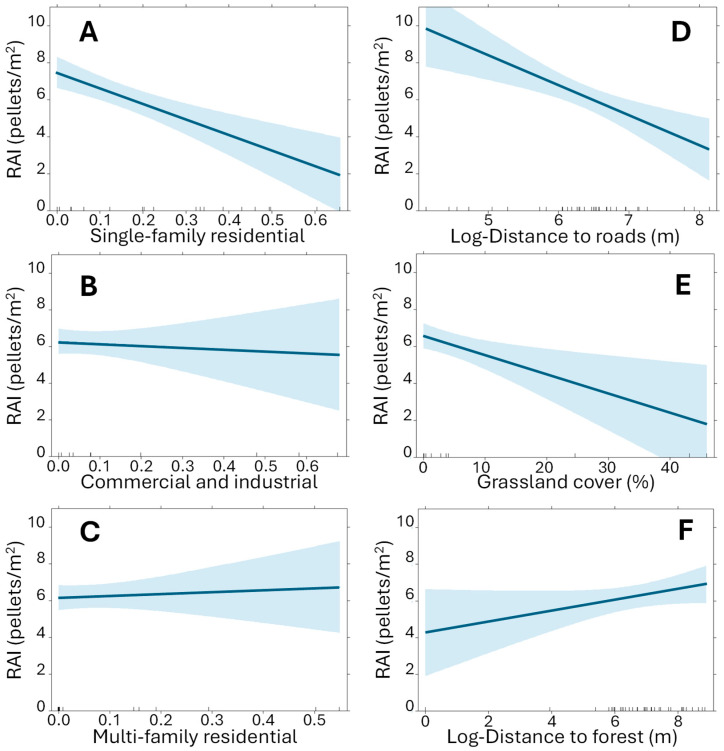
Relationships between the rabbit abundance index (RAI) and informative variables of hypotheses regarding ‘Human activities’ (urbanized cover **A**–**C**; and distance to transport infrastructures **D**), ‘Vegetation cover within the rabbit home range’ (grasslands **E**) and ‘Larger landscape-scale factors’ (distance to forest patches **F**), according to the final selected model. The solid lines represent the estimated relationships according to the final model, and funnel plots their 95% confidence intervals. Detailed explanations of variables can be found in the text and Table 2.

In the construction of the final model (AIC = 154.2), it was found that the type of urban planning is relevant to explain the abundance of rabbits, obtaining a highly significant model (F_6,29_ = 6.05; *p* = 0.0003) that explains somewhat over 46% of the variability observed in the data (adjusted R^2^ = 0.464). According to this model (Figure 2A–D, Table 3), rabbit abundance responds strongly to two factors associated with human activity: urban cover and proximity to major transportation infrastructure (mainly roads in our case). In relation to urban occupation, it has been found that single-family residential areas significantly reduce rabbit abundance, while commercial or industrial areas, and multi-family buildings, have virtually no effect. Furthermore, proximity to transportation infrastructure is associated with a highly significant increase in the abundance of the species. At the same time, there is a highly significant effect of grassland cover within the home range, which is associated with a decrease in rabbit abundance (Figure 2E). Finally, the distance to forest patches larger than 5 ha modulates the abundance of the species, which is slightly (and marginally significant) higher at greater distances from forest areas (Figure 2F).

## 4. Discussion

Our results show that rabbit abundance in peri-urban environments is associated with variables from the three proposed hypotheses, although human activity plays a fundamental role. However, human effects are more complex than initially hypothesized, since, although roadsides are confirmed as refuges for the species, the same is not true for urban areas, which in the case of single-family residential areas stand as negative for the species. Complementarily, the vegetation structure at the home range scale and the landscape context at a larger scale drive rabbit abundance, although the latter is less relevant. In short, the results define more explicitly the conditions under which human activity generates a refuge effect for rabbits and suggest that protection from potential predators (wild ones but also pets around gardens) may be key for the species in these areas.

### 4.1. Human Determinants of Rabbit Abundance

Firstly, this study highlights that urban occupation conditions the abundance of wild rabbits, but (i) it does so differentially depending on the type of urban development and (ii) the most relevant effect is negative and associated with the presence of single-family residential areas, contrary to the initial hypothesis of a generalized refuge effect of all urban environments.

The abundance of rabbits is drastically reduced around single-family residential areas, which may be associated with human disturbance and the constant presence of dogs and cats, both under human supervision and feral or free-ranging ones. The presence of people walking, often accompanied by dogs, in open areas around residential areas is very common and it is associated with new ways of living in cities [39,57]. Although the situation in peri-urban areas is less analyzed, it is known that the presence of visitors with dogs in natural areas modifies the spatial patterns of occupation by vertebrates [58,59] and, in the case of lagomorphs, abundance or activity is reduced close to frequented trails, both due to predation and fear [60,61]. At the same time, cats living in single-family housing developments often leave their homes and roam around the neighborhood [43], and it has been proven that free-ranging domestic cats are effective predators of rabbits [38,62]. Therefore, it is plausible that the abundance of rabbits in the vicinity of residential areas is reduced by a combination of disturbance and predation by free-ranging pets, and that this effect is more pronounced in single-family residential areas, where the existence of gardens facilitates dog ownership and cats wandering outside their homes. The presence of some wild predators in gardens adds to this, like Eagle owl (*Bubo bubo*), Booted eagle (*Hieraaetus pennatus*) or kites (*Milvus milvus*, *M. migrans*) known to nest in large gardens in Central Spain and hunt in these areas [63,64]. Such combination may thus further reduce rabbit populations either by predation or fear [65].

On the contrary, this effect is diluted in other types of urban development, and it could even be reversed in the case of dense urban sites, according to the (not significantly different from zero) estimators of the final model. This effect could be due to a lower number of walkers in the backyards of industrial areas and/or fewer feral and uncontrolled pets around multi-dwelling blocks. In fact, exercise and leisure walking are more frequent in peri-urban areas while directional walking (e.g., to retail stores) is more frequent in urban areas [66]. In this sense, the use of city gardens to walk with dogs is most common in dense neighborhoods, while empty yards around single-family residential areas (often accessible through dirt paths) are selected by their inhabitants to walk, and dogs go frequently unleashed there [39,42]. Moreover, cats in multi-dwelling blocks most often have their movements restricted to inside the apartments [43].

Secondly, it is confirmed that the presence of transport infrastructure facilitates the development of dense rabbit populations in their proximities [32,67], at least in areas subject to intense human activity (see however [68] for forest areas). The abundance of rabbits in the vicinity of roads is due to a combination of effects at different scales, with both elements of the landscape matrix at a kilometric scale and of road verge structure being decisive factors [30,31]. In this sense, our work complements existing information for small and medium scales, as it verifies on a regional scale the strong positive effect of infrastructures on rabbit abundance, supported by sampling along a gradient in the range of hundreds of meters from the roads, and controlling for other landscape effects. This reinforces the generality of patterns found in regional analyses in which the explanatory landscape variables were measured in 1 km [31] and 10 km [32] cell grids, further refining others that only worked with points located beside the infrastructure or at fixed distances from it (e.g., [30,68]). The reasons for this pervasive high abundance of rabbits close to transport infrastructures have been linked to the presence of undisturbed soil where they can establish burrows, more constant availability of food, and/or reduced rates of natural predation, which may compensate for increased mortality due to roadkill [40,69].

### 4.2. Landscape Determinants of Rabbit Abundance

In relation to the effects of the surrounding vegetation, the negative effect on rabbit abundance of grassland cover at the home range scale stands out, contrary to the trends most frequently described in the literature. Most studies on this topic show a positive relationship between rabbit abundance and grassland cover or other open areas [24,30], generally in the form of grassland–scrub ecotones [26,49]. On the contrary, in our case a negative association with grassland cover was detected, even though grass cover was small at most sampling points (average 3.5%). However, given the low or no grazing of the sampled land, the cover of nitrophilous grass under the scrub is high, and it should provide more than enough food for rabbits. In this context, it is possible that the provision of shelter from predators (or chasing dogs) is more decisive for rabbits in plots that also have a significant amount of bare soil (average 12.5%), and that this is reflected in this negative association with grasslands (see also [26,70]).

Finally, the landscape-scale effect detected for forest patches may be associated with the presence of wild predators, which in urbanized areas seek refuge in forest patches and venture out from there into open areas to hunt. This is reasonable given that forests are the preferred habitat of numerous mesocarnivores, both for feeding and for sheltering [53,71,72], even though many predators are capable of moving around and hunting in more anthropized landscapes. These forest patches are also a common refuge for raptors in landscapes heavily impacted by humans (see above). In short, knowledge about predation rates in peri-urban areas is still scarce [41], proximity to forest remnants may increase it, and it is precisely predation rates that may be decisive in determining rabbit population dynamics in these areas.

### 4.3. Final Remarks

Finally, it should be noted that the social perception that there is a large abundance of rabbits in peri-urban environments could be biased by the fact that the species is easier to observe in these areas. Species with high behavioral flexibility show a greater predisposition to colonize urban areas, and this may be happening with rabbits [73]. The behavioral plasticity of rabbits would allow exposure to humans during their growth to result in a tame behavior in those populations that are in more frequent and direct contact with humans, around villages [74]. This would differentiate them from rabbits grown in wilder environments, making them more frequently observable even during the day [12,75]. This difference in behavior would be responsible for a decoupling between human perception and biological reality; a fact observed in various situations [76,77,78]. Another plausible explanation for this fact would be that the slight (non-significant) trend detected in our data, pointing to denser populations of rabbits in multi-dwelling block sites, indicates the beginning of a gradient not fully sampled. In such a case, rabbits could establish very dense populations in more purely urban areas. Thus, although sampling covered a wide variety of situations along the rural–urban gradient, the number of samples located beside high-density urban areas was small; and it is possible that it is precisely in these places where citizens are surprised by the presence of rabbits in parks, roundabouts, and streets [69].

## 5. Conclusions

From an applied perspective, the results highlight the potential relevance of peri-urban areas for the settlement of wild animal populations. In the case of the European rabbit, classified as endangered [25], the results show that peri-urban areas (i) can maintain significant populations of the species, even though they do not constitute effective refuges for them. Moreover, (ii) it is also possible that, linked to these rabbit populations, some species of raptors (Eagle owl, Booted Eagle, Iberian Imperial Eagle) or carnivores tolerant of humans (Red Fox, Stone Marten, etc.) may in turn maintain certain population pockets (or dispersing juveniles) in areas subject to intense human use. In fact, our results show that variables associated with human use are the most explanatory of rabbit abundance in this densely populated region. In this situation, the connectivity with less disturbed areas, the existence of Green Infrastructure elements improving it, or the proximity to protected natural sites can reinforce the interest in conserving peri-urban environments in these regions where land sparing strategies for conservation are unfeasible [10].

## Figures and Tables

**Table 1:** System used to estimate the abundance of rabbit pellets in transects. In each 1-m^2^ grid cell, a visual estimation of pellet abundance was made in 5 classes, corresponding to the pellet ranges indicated in the second row. For the computation of the abundance index, the estimated number of pellets in each grid cell was replaced in the summatory by the median value for that range (Ci).

Class	0	1	2	3	4	5
Pellet range	0	1–2	3–15	16–50	51–150	If >150, estimated hundred range (e.g., 151–250, 251–350, 351–450…)
Ci	0	1.5	9	33	100	The median value (200, 300, 400…)

**Table 2:** Variables used to characterize the urbanized area and vegetation cover in the 300 m buffer defined around each sampling site.

Variable Name	Description	Source
Urbanized area		
Single-family residential	Residential areas composed of single-family houses with gardens	Visual categorization on 2021 aerial images and area measurements performed in GIS
Commercial or industrial	Industrial and commercial areas	
Multi-family residential	Residential areas of multi-dwelling blocks	
Vegetation of buffer surfaces not urbanized		
Forest	Natural forest and tree plantations	Categories reclassified from [50] and checked on aerial images taken in 2021. Surfaces measured with GIS
Shrubland	Shrublands with grass cover underneath	
Grassland	Open grassland without shrub cover	
Woody crops	Vineyards, olive, almond, or other tree crops	
Herbaceous crops	Mainly cereal and fodder crops, including fallow land	
Barren	Areas without plant cover	

**Table 3:** Results of the final model describing the relative abundance of rabbits in terms of relevant explanatory variables. Detailed explanations of variables can be found in Table 2. Probabilities (*p*) in the table are accompanied by their statistical significance: *** < 0.001; ** < 0.01; · < 0.1.

Variable	Estimate ± SE	F-Value	*p*
Intercept	16.203 ± 3.241	24.98	<0.0001 ***
Distance to transport infrastructures	−1.624 ± 0.425	14.61	<0.0001 ***
Urbanized: single-family residential	−8.388 ± 1.840	20.77	<0.0001 ***
Urbanized: commercial or industrial	−1.072 ± 2.354	0.21	0.652
Urbanized: multi-family residential	1.046 ± 2.362	0.20	0.661
Grassland cover (%)	−0.103 ± 0.036	8.39	0.0071 **
Distance to forest patches ≥ 5 ha	0.295 ± 0.169	3.05	0.0915 ·

## Data Availability

The basic dataset of the paper is available at the Open Free Digital Repository e-cienciaDatos (https://doi.org/10.21950/CCNJ2H).

## Abbreviations

The following abbreviations are used in this manuscript:

RAI	Rabbit Abundance Index
AIC	Akaike’s Information Criterium
